# Supplementary material for: Cellular and subcellular specialization enables biology-constrained deep learning
Source: bioRxiv. 2025 May 27:2025.05.22.655599. Preprint. [Version 1] doi: 10.1101/2025.05.22.655599 (PMC12154792; doi:10.1101/2025.05.22.655599)
Supplement: Supplement 1 [file NIHPP2025.05.22.655599v1-supplement-1.pdf]

bioRxiv preprint doi: https://doi.org/10.1101/2025.05.22.655599; this version posted May 27, 2025. The copyright holder for this preprint (which was not certified by peer review) is the author/funder, who has granted bioRxiv a license to display the preprint in perpetuity. It is made available under aCC-BY 4.0 International license.

Supplementary Figures

Supplementary Figure S1.

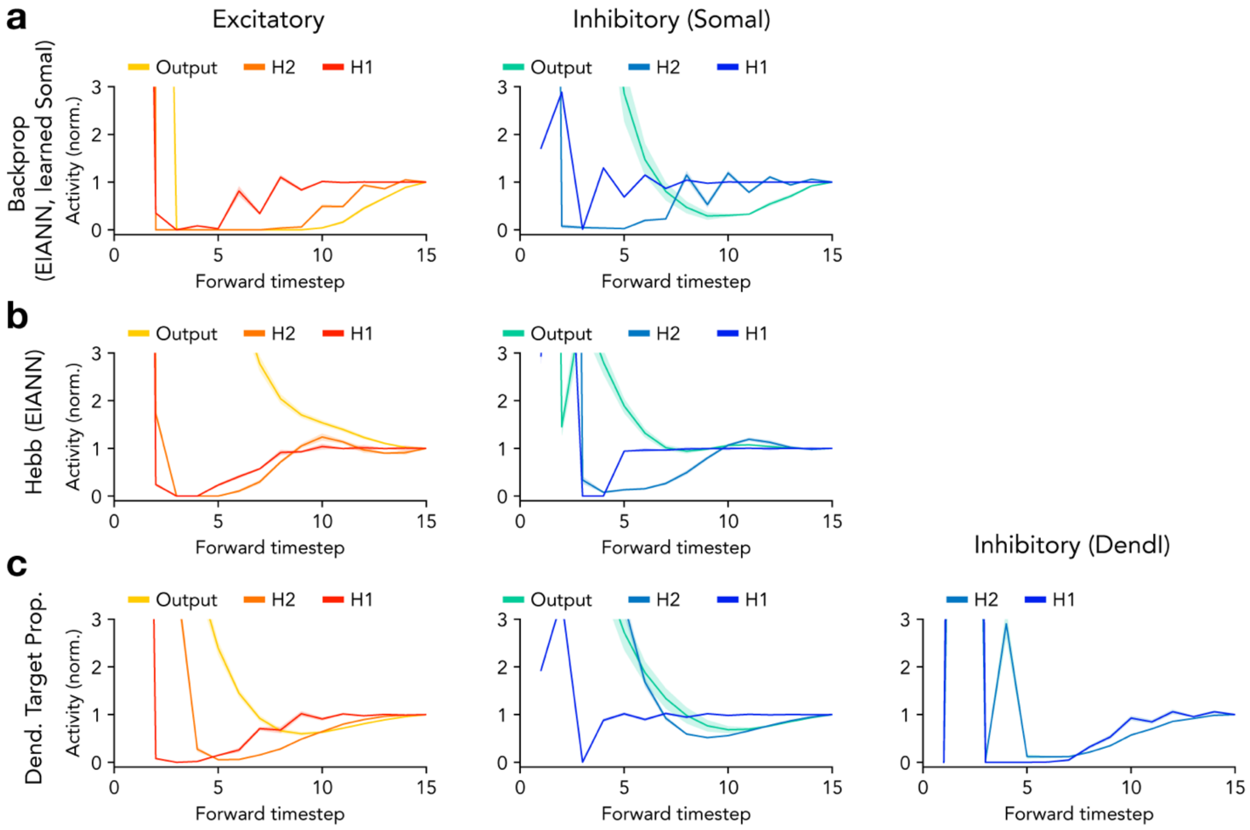

Supplementary Figure S1. Equilibration dynamics of neuronal activity in recurrent EIANNs.
Graphs show the dynamics of neuronal activity across 15 time steps of equilibration. To compare neuronal subpopulations, the average activity of each population is normalized such that its final value at the end of equilibration is one. Large onset transients are truncated for display purposes. *Left*: excitatory neurons; *middle*: soma-targeting inhibitory neurons (Somal); *right*: dendrite-targeting inhibitory neurons (Dendl). **a,** EIANN trained with backpropagation. **b,** EIANN trained with the normalized Hebbian learning rule. **c,** Dendritic EIANN trained with *dendritic target propagation*. **a-c**, Shading indicates standard deviation across five instances of each network.

bioRxiv preprint doi: https://doi.org/10.1101/2025.05.22.655599; this version posted May 27, 2025. The copyright holder for this preprint (which was not certified by peer review) is the author/funder, who has granted bioRxiv a license to display the preprint in perpetuity. It is made available under aCC-BY 4.0 International license.

Supplementary Figure S2.

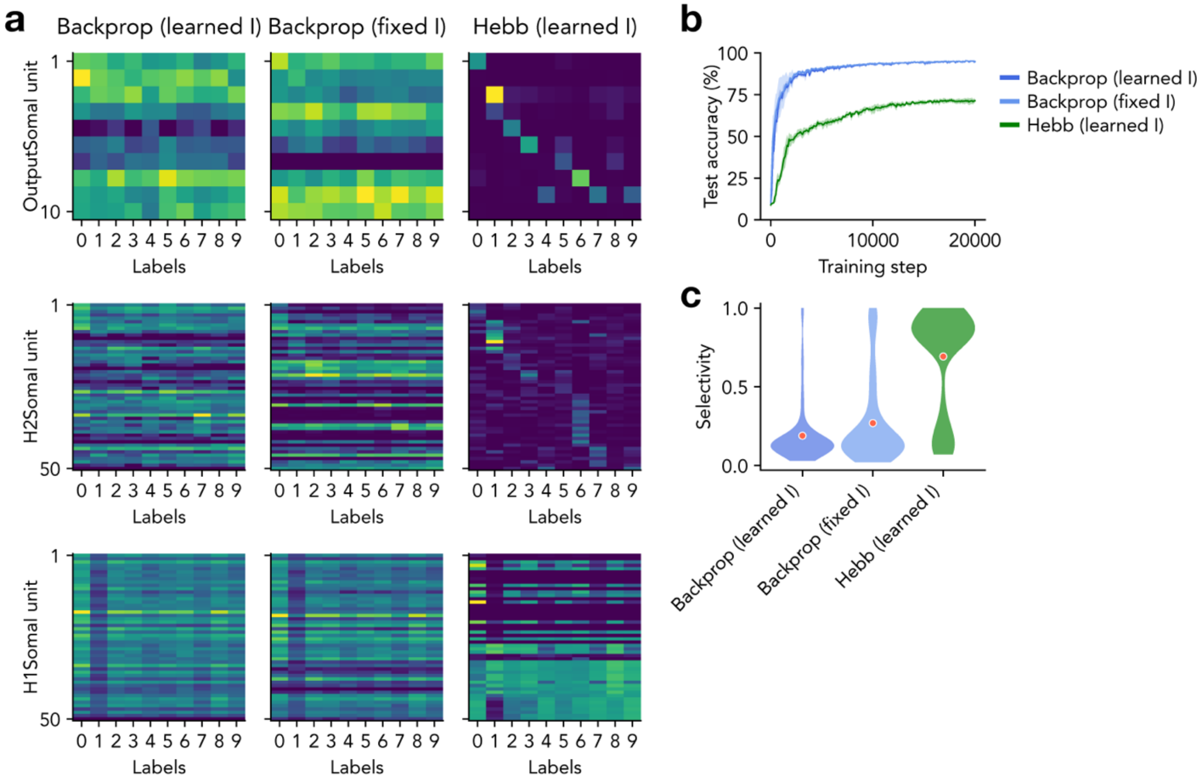

Supplementary Figure S2. Plasticity of somatic inhibition is not required for image classification.
a, Average neuronal population activity in response to each class of handwritten digits. *Left*: Shown are soma-targeting inhibitory neurons (Somal) from the Output layer (10 units, top), layer H2 (50 units, middle), and layer H1 (50 units, bottom) from a recurrent EIANN trained with backpropagation with learned connections to and from Somal units. *Middle*: Same as *left* for a recurrent EIANN trained with backpropagation with connections to and from Somal units that are fixed at initialization and not learned. *Right*: same as *left* for a recurrent EIANN trained with a normalized Hebbian learning rule. **b,** Classification performance accuracy. Shading indicates standard deviation across five instances of each network. **c,** Selectivity of Somal units (in all layers) over stimulus classes.

bioRxiv preprint doi: https://doi.org/10.1101/2025.05.22.655599; this version posted May 27, 2025. The copyright holder for this preprint (which was not certified by peer review) is the author/funder, who has granted bioRxiv a license to display the preprint in perpetuity. It is made available under aCC-BY 4.0 International license.

Supplementary Figure S3.

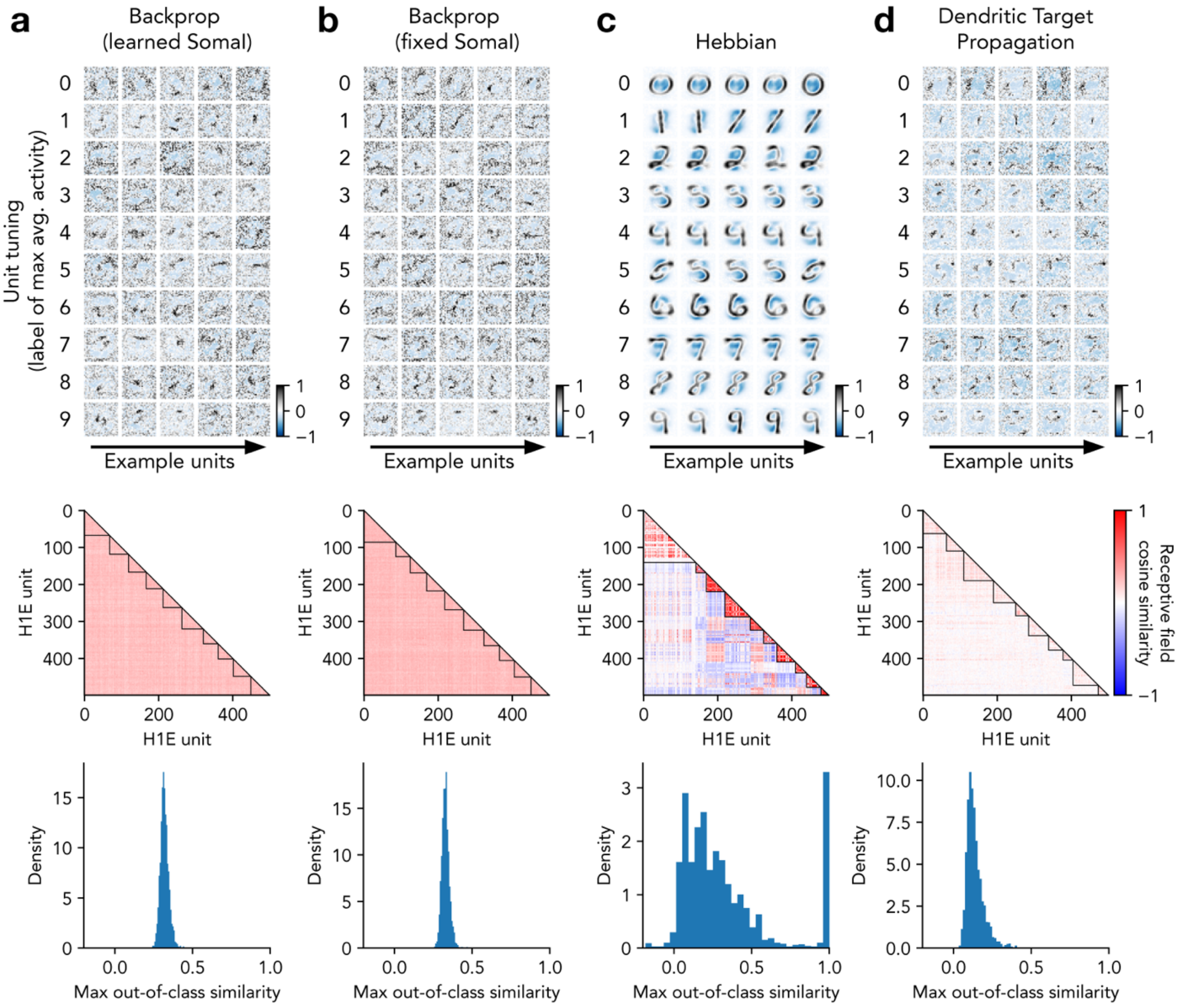

Supplementary Figure S3. Analysis of hidden layer neuronal receptive fields in EIANNs.

a, Receptive fields are analyzed from H1E units from a recurrent EIANN trained with backpropagation with learned connections to and from Somal units. *Top*: Example receptive fields. Row labels indicate the class that produces the largest activity (averaged across samples) for each unit. *Middle*: Heatmaps depict the cosine similarity of receptive fields between pairs of H1E units. Units are sorted by their preferred stimulus class, and black lines demarcate groups of units that share a class preference. *Bottom*: Histograms show, for each unit, the maximum cosine similarity compared to other units that do not share the same stimulus class preference. **b,** Same as **a** for a recurrent EIANN trained with backpropagation with connections to and from Somal units that are fixed at initialization and not learned. **c,** same as **a** for a recurrent EIANN trained with a normalized Hebbian learning rule. **d,** same as **a** for a dendritic EIANN trained with *dendritic target propagation*.

bioRxiv preprint doi: https://doi.org/10.1101/2025.05.22.655599; this version posted May 27, 2025. The copyright holder for this preprint (which was not certified by peer review) is the author/funder, who has granted bioRxiv a license to display the preprint in perpetuity. It is made available under aCC-BY 4.0 International license.

Supplementary Figure S4.

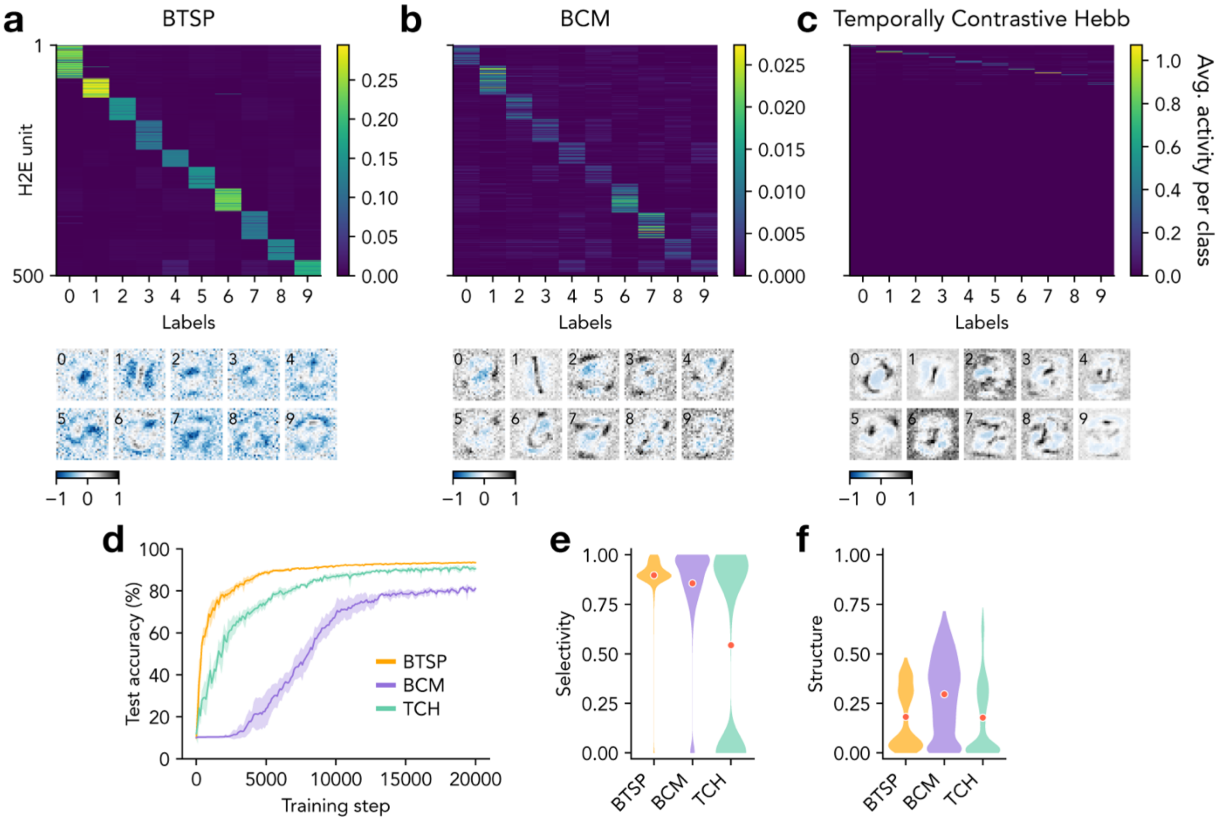

Supplementary Figure S4. Excitatory neuron selectivity in dendritic EIANNs trained with biological learning rules.

a, *Top*: Average neuronal population activity in response to each class of handwritten digits. Shown are 500 excitatory (E) units in the second hidden layer (H2) from a dendritic EIANN trained with *dendritic target propagation* and using the BTSP learning rule to learn bottom-up excitatory weights. *Bottom:* Example receptive fields selected from H2E units. Numbers in the top left corners indicate the label of the class that produces the largest activity (averaged across samples) for each unit. **b,** Same as **a** for a dendritic EIANN using the BCM rule to learn bottom-up excitatory weights. **c,** Same as **a** for a dendritic EIANN using the TCH rule to learn bottom-up excitatory weights. **d,** Classification performance accuracy. Shading indicates standard deviation across five instances of each network. **e,** Selectivity of E units (in both hidden layers) over stimulus classes. **f,** Spatial structure of the receptive fields of E units (in both hidden layers), as measured by spatial autocorrelation (Moran's I).

bioRxiv preprint doi: https://doi.org/10.1101/2025.05.22.655599; this version posted May 27, 2025. The copyright holder for this preprint (which was not certified by peer review) is the author/funder, who has granted bioRxiv a license to display the preprint in perpetuity. It is made available under aCC-BY 4.0 International license.

Supplementary Figure S5.

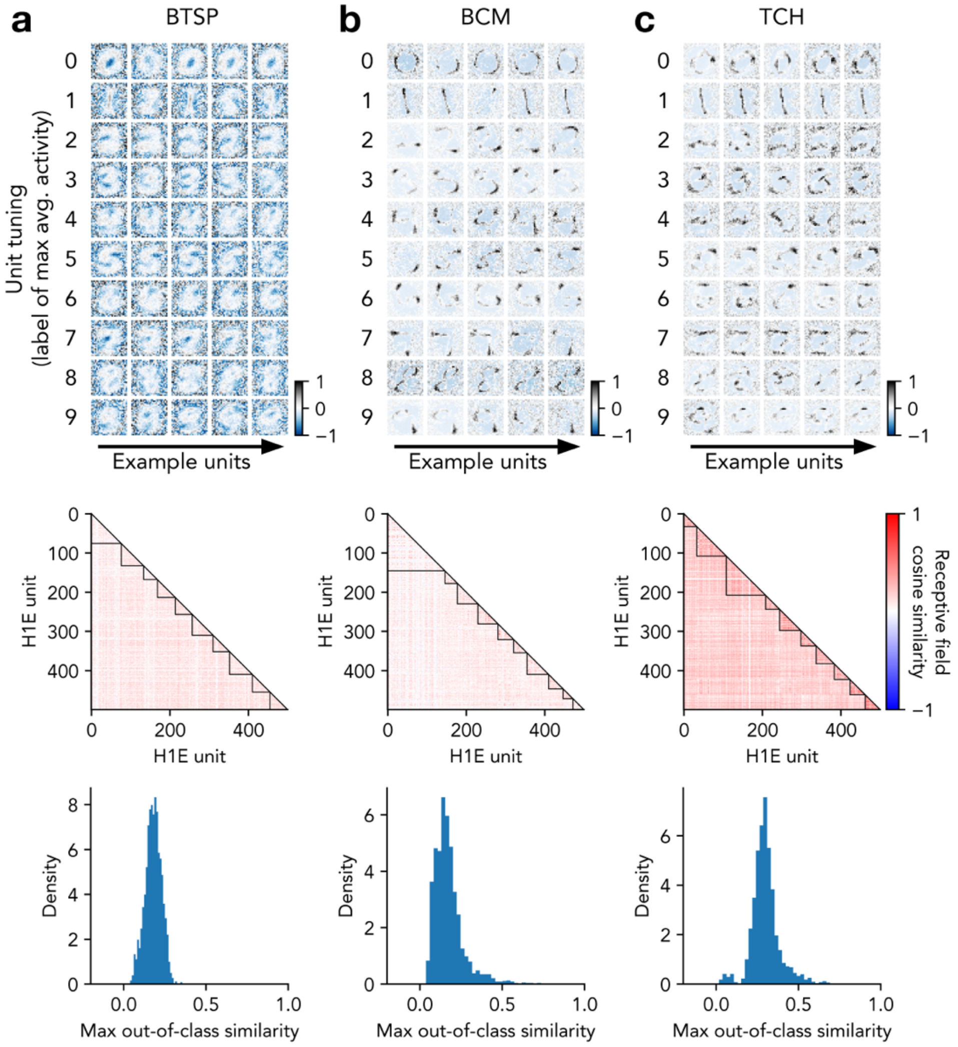

Supplementary Figure S5. Analysis of hidden layer neuronal receptive fields in dendritic EIANNs trained with biological learning rules.

a, Receptive fields are analyzed from H1E units from a dendritic EIANN trained with *dendritic target propagation* and using the BTSP learning rule to learn bottom-up excitatory weights. *Top*: Example receptive fields. Row labels indicate the class that produces the largest activity (averaged across samples) for each unit. *Middle*: Heatmaps depict the cosine similarity of receptive fields between pairs of H1E units. Units are sorted by their preferred stimulus class, and black lines demarcate groups of units that share a class preference. *Bottom*: Histograms show, for each unit, the maximum cosine similarity compared to other units that do not share the same stimulus class preference. **b,** Same as **a** for a dendritic EIANN using the BCM rule to learn bottom-up excitatory weights. **c,** Same as **a** for a dendritic EIANN using the TCH rule to learn bottom-up excitatory weights.

bioRxiv preprint doi: https://doi.org/10.1101/2025.05.22.655599; this version posted May 27, 2025. The copyright holder for this preprint (which was not certified by peer review) is the author/funder, who has granted bioRxiv a license to display the preprint in perpetuity. It is made available under aCC-BY 4.0 International license.

Supplementary Table S1.

	Architecture	Hidden Layers	Algorithm	W Learning Rule	B Learning Rule	Bias	MNIST Accuracy (20k samples)	MNIST Accuracy (50k samples)
**Feedforward ANN**	ANN	2	Backprop	Gradient descent	Symmetric ($B = W^T$)	Zero	95.48 ± 0.20	95.48 ± 0.20
**Feedforward ANN (fixed hidden)**	ANN	2	Backprop	Gradient descent	Symmetric ($B = W^T$)	Zero	87.73 ± 0.08	89.68 ± 0.24
**Feedforward ANN (no hidden)**	ANN	0	Backprop	Gradient descent	Symmetric ($B = W^T$)	Zero	89.43 ± 0.08	90.56 ± 0.05
**Backprop (fixed Somal)**	EIANN	2	Backprop	Gradient descent	Symmetric ($B = W^T$)	Zero	94.70 ± 0.38	96.38 ± 0.16
**Backprop (learned Somal)**	EIANN	2	Backprop	Gradient descent	Symmetric ($B = W^T$)	Zero	94.76 ± 0.27	96.53 ± 0.10
**Backprop (no Somal)**	EIANN	2	Backprop	Gradient descent	Symmetric ($B = W^T$)	Zero	60.65 ± 1.72	64.01 ± 1.03
**Hebbian**	EIANN	2	Unsupervised (no propagation)	Hebb + Weight Norm.	Symmetric ($B = W^T$)	Zero	71.35 ± 0.64	73.24 ± 0.60
**Fixed DendI (random)**	Dend EIANN	2	Dend Target Prop	LDS	Symmetric ($B = W^T$)	Zero	86.80 ± 0.50	87.36 ± 0.59
**Learned DendI (local backprop)**	Dend EIANN	2	Dend Target Prop + Backprop (DendI)	LDS	Symmetric ($B = W^T$)	Zero	94.08 ± 0.11	95.04 ± 0.20
**Learned DendI (Hebb)**	Dend EIANN	2	Dend Target Prop	LDS	Symmetric ($B = W^T$)	Zero	92.62 ± 0.10	94.29 ± 0.13
**Temporally Contrastive Hebb**	Dend EIANN	2	Dend Target Prop	TCH	Symmetric ($B = W^T$)	Zero	90.55 ± 0.30	90.89 ± 0.68
**BCM**	Dend EIANN	2	Dend Target Prop	BCM	Symmetric ($B = W^T$)	Zero	81.10 ± 0.52	68.92 ± 0.89
**BTSP**	Dend EIANN	2	Dend Target Prop	BTSP	Symmetric ($B = W^T$)	Zero	93.43 ± 0.10	94.08 ± 0.13
**LDS, fixed top-down**	Dend EIANN	2	Dend Target Prop	LDS	Fixed random	Zero	90.93 ± 0.33	92.86 ± 0.19
**LDS, learned top-down**	Dend EIANN	2	Dend Target Prop	LDS	TCH + Weight Norm.	Zero	92.47 ± 0.13	93.96 ± 0.12
**BTSP, fixed top-down**	Dend EIANN	2	Dend Target Prop	BTSP	Fixed random	Zero	84.24 ± 0.47	83.32 ± 0.47
**BTSP, learned top-down**	Dend EIANN	2	Dend Target Prop	BTSP	TCH + Weight Norm.	Zero	91.65 ± 0.17	93.50 ± 0.14

Supplementary Table S1. Handwritten digit classification performance accuracy.

bioRxiv preprint doi: https://doi.org/10.1101/2025.05.22.655599; this version posted May 27, 2025. The copyright holder for this preprint (which was not certified by peer review) is the author/funder, who has granted bioRxiv a license to display the preprint in perpetuity. It is made available under aCC-BY 4.0 International license.

Supplementary Table S2.

	Architecture	Hidden Layers	Algorithm	W Learning Rule	B Learning Rule	Bias	Spiral Accuracy (1 epoch)	Spiral Accuracy (10 epochs)
**Feedforward ANN (no hidden)**	ANN	0	Backprop	Gradient Descent	Symmetric ($B = W^T$)	Learned	55.35 ± 2.64	51.38 ± 2.17
**Feedforward ANN (learned bias)**	ANN	2	Backprop	Gradient Descent	Symmetric ($B = W^T$)	Learned	95.77 ± 0.41	97.82 ± 0.10
**Feedforward ANN (no bias)**	ANN	2	Backprop	Gradient Descent	Symmetric ($B = W^T$)	Zero	48.95 ± 0.07	48.58 ± 0.16
**Backprop (EIANN)**	EIANN	2	Backprop	Gradient Descent	Symmetric ($B = W^T$)	Learned	95.87 ± 0.27	97.08 ± 0.35
**Dendritic Target Propagation**	Dend EIANN	2	Dend Target Prop	LDS	Symmetric ($B = W^T$)	Learned	91.87 ± 0.74	90.13 ± 1.04

Supplementary Table S2. Two-dimensional spiral pattern classification performance accuracy.

bioRxiv preprint doi: https://doi.org/10.1101/2025.05.22.655599; this version posted May 27, 2025. The copyright holder for this preprint (which was not certified by peer review) is the author/funder, who has granted bioRxiv a license to display the preprint in perpetuity. It is made available under aCC-BY 4.0 International license.

Supplementary Table S3.

Param name	Feedforward ANN	Feedforward ANN (fixed hidden)	Feedforward ANN (no hidden)	Backprop (fixed Somal)	Backprop (learned Somal)	Backprop (no Somal)	Hebbian
W_{init} (H1)	2.9573	5.0120	-	2.4169	2.3418	0.0313	-
η, W (H1)	0.1652	-	-	0.3981	0.3913	0.1904	0.0055
W_{init} (H2)	0.3069	0.3272	-	0.5428	0.7159	1.4197	-
η, W (H2)	0.1652	-	-	0.3981	0.3913	0.1904	0.0055
W_{init} (Output)	2.0936	0.0203	0.0265	3.2817	1.0173	3.9650	1.1091
η, W (Output)	0.0217	0.0582	0.0041	0.0708	0.3265	0.0110	0.0193
Y_{init} (Somal, H1)	-	-	-	1.1159	1.5332	-	-
W_{init} (Somal, H1)	-	-	-	3.1371	2.0286	-	-
Q_{init} (Somal, H1)	-	-	-	0.4836	0.3540	-	-
R_{init} (Somal, H1)	-	-	-	1.2959	1.2781	-	-
Y_{init} (Somal, H2)	-	-	-	0.4713	0.9795	-	-
W_{init} (Somal, H2)	-	-	-	1.4413	1.0560	-	-
Q_{init} (Somal, H2)	-	-	-	1.5897	0.1488	-	-
R_{init} (Somal, H2)	-	-	-	2.4085	1.4983	-	-
Y_{init} (Somal, Output)	-	-	-	1.5076	0.9429	-	-
W_{init} (Somal, Output)	-	-	-	2.9499	0.8592	-	-
Q_{init} (Somal, Output)	-	-	-	0.5984	0.2251	-	-
R_{init} (Somal, Output)	-	-	-	1.5326	0.8824	-	-
η, Y (Somal, H1)	-	-	-	-	0.0016	-	0.0388
η, W (Somal, H1)	-	-	-	-	0.0851	-	0.0007
η, Q (Somal, H1)	-	-	-	-	0.0851	-	0.0007
η, R (Somal, H1)	-	-	-	-	0.0029	-	0.0523
η, Y (Somal, H2)	-	-	-	-	0.0016	-	0.0388
η, W (Somal, H2)	-	-	-	-	0.0851	-	0.0007
η, Q (Somal, H2)	-	-	-	-	0.0851	-	0.0007
η, R (Somal, H2)	-	-	-	-	0.0029	-	0.0523
η, Y (Somal, Output)	-	-	-	-	0.0016	-	0.0388
η, W (Somal, Output)	-	-	-	-	0.0851	-	0.0007
η, Q (Somal, Output)	-	-	-	-	0.0851	-	0.0007
η, R (Somal, Output)	-	-	-	-	0.0029	-	0.0523
W_{sum} (H1)	-	-	-	-	-	-	9.6360
Y_{sum} (Somal, H1)	-	-	-	-	-	-	5.6391
W_{sum} (Somal, H1)	-	-	-	-	-	-	13.5545
Q_{sum} (Somal, H1)	-	-	-	-	-	-	14.2162
R_{sum} (Somal, H1)	-	-	-	-	-	-	5.7558
W_{sum} (H2)	-	-	-	-	-	-	6.9501
Y_{sum} (Somal, H2)	-	-	-	-	-	-	2.4875
W_{sum} (Somal, H2)	-	-	-	-	-	-	6.1176
Q_{sum} (Somal, H2)	-	-	-	-	-	-	7.3201
R_{sum} (Somal, H2)	-	-	-	-	-	-	2.6716
Y_{sum} (Somal, Output)	-	-	-	-	-	-	0.1380
W_{sum} (Somal, Output)	-	-	-	-	-	-	5.5863
Q_{sum} (Somal, Output)	-	-	-	-	-	-	1.0857
R_{sum} (Somal, Output)	-	-	-	-	-	-	2.8848

Supplementary Table S3. Hyperparameters for networks trained on handwritten digit classification (Part 1).

bioRxiv preprint doi: https://doi.org/10.1101/2025.05.22.655599; this version posted May 27, 2025. The copyright holder for this preprint (which was not certified by peer review) is the author/funder, who has granted bioRxiv a license to display the preprint in perpetuity. It is made available under aCC-BY 4.0 International license.

Supplementary Table S4.

Param name	Fixed Dendl (random)	Learned Dendl (local backprop)	Learned Dendl (Hebb)	Temporally Contrastive Hebb	BCM
W_{init} (H1)	0.4807	1.7767	2.1944	0.6845	1.5778
η, W (H1)	0.0024	0.0055	0.0150	0.0357	0.0053
Y_{init} (Somal, H1)	1.3259	1.1354	1.1276	0.5709	0.7548
Y_{init} (Dendl, H1)	1.0422	1.0818	0.3087	1.1628	0.1169
η, Y (Dendl, H1)	0.1258	0.1326	0.0999	0.2148	0.0485
B_{scale} (H1)	3.1656	0.2206	0.4983	0.0983	0.4654
W_{init} (Somal, H1)	0.5832	1.9747	2.9554	0.6062	1.5437
Q_{init} (Somal, H1)	0.4593	0.3160	0.7922	0.2994	0.9798
R_{init} (Somal, H1)	1.1412	1.0789	1.4660	0.3530	0.5322
Q_{init} (Dendl, H1)	0.6163	1.1146	-	-	-
R_{init} (Dendl, H1)	1.4707	1.1525	-	-	-
W_{init} (H2)	1.3195	1.8245	0.1970	0.1834	0.0406
η, W (H2)	0.0024	0.0055	0.0150	0.0357	0.0053
Y_{init} (Somal, H2)	0.8653	0.4607	0.0503	0.3143	0.1938
Y_{init} (Dendl, H2)	0.5942	0.5361	1.6269	8.6285	0.8409
η, Y (Dendl, H2)	0.1258	0.1326	0.0999	0.2148	0.0485
B_{scale} (H2)	0.1113	16.3818	3.5503	1.8202	1.3422
W_{init} (Somal, H2)	1.6739	2.8770	0.1287	1.0033	0.2265
Q_{init} (Somal, H2)	0.5671	0.5031	0.7245	6.1742	0.5136
R_{init} (Somal, H2)	1.1483	0.9886	0.1518	1.5656	0.8107
Q_{init} (Dendl, H2)	4.1364	0.2039	-	-	-
R_{init} (Dendl, H2)	1.3141	1.1754	-	-	-
W_{init} (Output)	2.6245	0.3041	5.5741	3.8974	1.7049
η, W (Output)	0.0110	0.0008	0.0258	0.0027	0.0021
Y_{init} (Somal, Output)	1.0616	0.7467	0.1078	0.0599	0.0324
W_{init} (Somal, Output)	1.1392	0.5392	0.4388	0.1603	0.5259
Q_{init} (Somal, Output)	0.3536	2.2788	2.7491	0.3028	5.9660
R_{init} (Somal, Output)	0.2708	1.9147	2.5391	0.4902	1.1313
η, Q (Dendl, H1)	-	0.7095	0.0193	0.0684	0.0118
η, R (Dendl, H1)	-	0.0953	0.0152	0.0154	0.0234
η, Q (Dendl, H2)	-	0.7095	0.0193	0.0684	0.0118
η, R (Dendl, H2)	-	0.0953	0.0152	0.0154	0.0234
Q_{sum} (Dendl, H1)	-	-	15.8736	31.0697	4.0065
R_{sum} (Dendl, H1)	-	-	4.9424	2.1420	2.2479
Q_{sum} (Dendl, H2)	-	-	11.7313	10.7920	3.0778
R_{sum} (Dendl, H2)	-	-	4.3829	2.4900	2.2502
τ_θ, (H1)	-	-	-	-	14.4700
BCM k, (H1)	-	-	-	-	0.0215
τ_θ, (H2)	-	-	-	-	14.4700
BCM k, (H2)	-	-	-	-	0.0215
τ_θ, (Output)	-	-	-	-	21.6776
BCM k, (Output)	-	-	-	-	0.1594

Supplementary Table S4. Hyperparameters for networks trained on handwritten digit classification (Part 2).

bioRxiv preprint doi: https://doi.org/10.1101/2025.05.22.655599; this version posted May 27, 2025. The copyright holder for this preprint (which was not certified by peer review) is the author/funder, who has granted bioRxiv a license to display the preprint in perpetuity. It is made available under aCC-BY 4.0 International license.

Supplementary Table S5.

Param name	BTSP	LDS, fixed top-down	LDS, learned top-down	BTSP, fixed top-down	BTSP, learned top-down
W_{init} (H1)	3.5237	1.5073	1.3345	3.9821	3.6173
η, W (H1)	0.0101	0.0171	0.0178	0.0025	0.0080
BTSP λ, (H1)	0.0162	-	-	0.0158	0.0368
BTSP W_{max}, (H1)	0.3520	-	-	0.3067	0.3848
Y_{init} (Somal, H1)	2.1960	0.7136	1.1995	0.9458	0.9531
Y_{init} (Dendl, H1)	1.4309	0.1059	0.9117	0.8045	0.6644
η, Y (Dendl, H1)	0.0540	0.0383	0.1806	0.0137	0.0320
B_{scale} (H1)	0.1534	-	-	-	-
W_{init} (Somal, H1)	2.2087	2.8738	0.9968	3.6431	2.9392
Q_{init} (Somal, H1)	0.7036	0.8390	0.3449	0.3386	0.3796
R_{init} (Somal, H1)	1.1848	1.2690	0.8168	0.8938	0.8945
η, Q (Dendl, H1)	0.1477	0.0859	0.0305	0.1545	0.0859
Q_{sum} (Dendl, H1)	22.1510	9.1341	21.4930	32.7122	40.8871
η, R (Dendl, H1)	0.0517	0.0054	0.0584	0.0280	0.1953
R_{sum} (Dendl, H1)	2.5624	0.0566	4.0984	1.6384	5.2394
W_{init} (H2)	0.8324	1.3261	0.7726	1.3294	1.8581
η, W (H2)	0.0101	0.0171	0.0178	0.0025	0.0080
BTSP λ, (H2)	0.0162	-	-	0.0158	0.0368
BTSP W_{max}, (H2)	0.4408	-	-	0.3840	0.4818
Y_{init} (Somal, H2)	0.2761	1.0827	0.5477	0.1948	0.2615
Y_{init} (Dendl, H2)	6.2109	1.0116	1.1691	4.7234	3.7733
η, Y (Dendl, H2)	0.0540	0.0383	0.1806	0.0137	0.0320
B_{scale} (H2)	5.9497	-	-	-	-
W_{init} (Somal, H2)	2.4756	1.0048	0.9279	2.5163	1.3713
Q_{init} (Somal, H2)	0.3042	0.0130	0.3694	0.4541	0.7330
R_{init} (Somal, H2)	1.0033	0.7750	0.8262	1.1843	1.0274
η, Q (Dendl, H2)	0.1477	0.0859	0.0305	0.1545	0.0859
Q_{sum} (Dendl, H2)	53.4596	17.2363	19.8207	49.7326	15.2392
η, R (Dendl, H2)	0.0517	0.0054	0.0584	0.0280	0.1953
R_{sum} (Dendl, H2)	2.5428	3.5820	4.4111	2.9325	2.2955
W_{init} (Output)	8.5974	0.4323	1.3278	12.6410	12.9520
η, W (Output)	0.0258	0.0222	0.0162	0.0291	0.0274
BTSP λ, (Output)	0.0162	-	-	0.0158	0.0368
BTSP W_{max}, (Output)	0.4353	-	-	0.5854	0.7402
Y_{init} (Somal, Output)	0.1893	0.2388	0.5080	0.2754	0.3090
W_{init} (Somal, Output)	1.1341	0.3644	0.2541	1.3436	0.8117
Q_{init} (Somal, Output)	0.1276	0.5277	0.0414	0.2703	1.6620
R_{init} (Somal, Output)	1.0732	0.5435	0.4426	2.9279	2.5709
B_{init} (H1)	-	0.0959	-	0.0133	-
B_{init} (H2)	-	1.1218	-	2.7066	-
η, B (H1)	-	-	0.0245	-	0.0216
B_{sum} (H1)	-	-	0.3539	-	1.0467
η, B (H2)	-	-	0.0336	-	0.0088
B_{sum} (H2)	-	-	0.4448	-	0.5107

Supplementary Table S5. Hyperparameters for networks trained on handwritten digit classification (Part 3).

bioRxiv preprint doi: https://doi.org/10.1101/2025.05.22.655599; this version posted May 27, 2025. The copyright holder for this preprint (which was not certified by peer review) is the author/funder, who has granted bioRxiv a license to display the preprint in perpetuity. It is made available under aCC-BY 4.0 International license.

Supplementary Table S6.

Param name	Feedforward ANN (no hidden)	Feedforward ANN (learned bias)	Feedforward ANN (no bias)	Backprop (EIANN)	Dendritic Target Propagation
W_{init} (Output)	5.3865	0.5434	3.5188	5.9778	5.4269
η, W (Output)	0.0816	0.0108	0.0013	0.6722	0.0977
W_{init} (H1)	-	3.0922	0.7662	2.3272	3.7442
η, W (H1)	-	0.2344	0.0087	0.1770	0.0856
W_{init} (H2)	-	1.0685	0.1340	5.1834	3.3963
η, W (H2)	-	0.2344	0.0087	0.1770	0.0856
Y_{init} (Somal, H1)	-	-	-	4.7081	1.7171
W_{init} (Somal, H1)	-	-	-	1.0646	8.5268
Q_{init} (Somal, H1)	-	-	-	0.7309	2.5573
R_{init} (Somal, H1)	-	-	-	1.9856	3.8213
Y_{init} (Somal, H2)	-	-	-	3.2740	1.9616
W_{init} (Somal, H2)	-	-	-	3.3866	3.3568
Q_{init} (Somal, H2)	-	-	-	0.6920	2.6728
R_{init} (Somal, H2)	-	-	-	1.6564	2.5278
Y_{init} (Somal, Output)	-	-	-	1.7630	1.1147
W_{init} (Somal, Output)	-	-	-	2.2899	1.9824
Q_{init} (Somal, Output)	-	-	-	1.4600	0.9623
R_{init} (Somal, Output)	-	-	-	1.5592	3.9245
Y_{init} (Dendl, H1)	-	-	-	-	0.7285
η, Y (Dendl, H1)	-	-	-	-	0.2102
B_{scale} (H1)	-	-	-	-	0.1879
η, Q (Dendl, H1)	-	-	-	-	0.0803
Q_{sum} (Dendl, H1)	-	-	-	-	9.4193
η, R (Dendl, H1)	-	-	-	-	0.0134
R_{sum} (Dendl, H1)	-	-	-	-	1.1712
Y_{init} (Dendl, H2)	-	-	-	-	2.7135
η, Y (Dendl, H2)	-	-	-	-	0.2102
B_{scale} (H2)	-	-	-	-	0.7150
η, Q (Dendl, H2)	-	-	-	-	0.0803
Q_{sum} (Dendl, H2)	-	-	-	-	11.6900
η, R (Dendl, H2)	-	-	-	-	0.0134
R_{sum} (Dendl, H2)	-	-	-	-	2.1474

Supplementary Table S6. Hyperparameters for networks trained on two-dimensional spiral pattern classification.
